# Influences of Nursing Professionalism, Empathy, and Clinical Decision-Making Ability on Shared Decision-Making Awareness among Hemodialysis Nurses

**DOI:** 10.1155/2024/2518065

**Published:** 2024-04-08

**Authors:** Junghwa Yun, Mihyeon Seong, Youngmi Cho, Sohyune Sok

**Affiliations:** ^1^Department of Nursing, Graduate School, Kyung Hee University, Seoul, Republic of Korea; ^2^DKMediinfo Nursing Information Research Institute, Changwon-si, Gyeongsangnam-do, Republic of Korea; ^3^Department of Nursing, Sun Moon University, Asan-si 31460, Chungcheongnam-do, Republic of Korea; ^4^College of Nursing Science, Kyung Hee University, Seoul, Republic of Korea

## Abstract

**Aim:**

To examine the relationships among nursing professionalism, empathy, and clinical decision-making ability and the factors influencing shared decision-making awareness in hemodialysis nurses.

**Background:**

Self-management and treatment for hemodialysis patients are essential for maintaining health and life in daily life. In this process, shared decision-making in which patients, nurses, and medical teams participate and make decisions together has a greater impact on the health recovery and improvement of quality of life for hemodialysis patients than for any other chronic disease patients.

**Methods:**

A cross-sectional descriptive design was employed. Participants were 145 nurses working in the hemodialysis centers at hospitals in Seoul and Gyeonggi-do. Measures included the general characteristics of study participants, nursing professionalism, empathy, clinical decision-making ability, and shared decision-making awareness. Data were collected from May to July, 2022, and multiple linear regression analysis was used to examine the predictive factors of shared decision-making awareness.

**Results:**

The strongest predictor was empathy, followed by clinical decision-making ability and the level of education. The explanatory power of the final regression model was 23%.

**Conclusions:**

Empathy towards hemodialysis patients was an important factor influencing the shared decision-making awareness in hemodialysis nurses. *Implications for Nursing Management*. In nursing management, nursing managers or nurses should pay attention to influencing factors to improve the shared decision-making awareness of hemodialysis nurses. Empathy towards hemodialysis patients need to be reinforced to improve the shared decision-making awareness of hemodialysis nurses.

## 1. Introduction

In hemodialysis, self-management such as regular dialysis, fluid and dietary restrictions, drug treatment, and vascular monitoring is essential for maintaining health and life in daily life.

Disease awareness and self-care management have a positive relationship [1], and in the case of hemodialysis patients, patients with positive disease awareness are compliant with treatment [2], and the disease perception of patients who applied the shared decision-making was relatively positive, and it was found to have a positive effect on clinical indicators and patient prognosis [3]. However, since the shared decision-making is influenced by the attitude of healthcare providers who lead the decision-making process, it is necessary to identify the shared decision-making awareness of medical personnel [4–6].

Shared decision-making is based on a shared mental model, which is the perception of, understanding of, or knowledge about a situation or process that is shared among team members through communication [7, 8]. The complexity and criticality of the current healthcare system require shared mental models to enhance safe and effective patient/client care [8, 9]. Each member's action can have an advantage in terms of efficiency, function, and strategy by using clear communication and guidance among team members in the shared decision-making process, and by sharing an understanding of goals and expectations towards a better quality of life for the patient [7, 9–11]. In a previous study [6], it was mentioned that the role of nurses in the shared decision-making process included being a health educator, spokesman, data collector, symptom and side effect manager, information sharer, and psychological supporter. Moreover, they said that nurses play a complementary role [5, 12] to doctors in the shared decision-making process and promote shared decision-making [13]. These characteristics are suitable for playing the role of a multidisciplinary coordinator as a member of a team in the shared decision-making process, and these allow nurses to integrate the biological and social life experiences of patients with chronic diseases in the shared decision-making process [9]. Since hemodialysis nurses spend several hours a week with long-term dialysis patients during treatment, a close therapeutic relationship can be formed [14, 15] and, thus, occupies a key position in terms of patient involvement [16, 17]. Likewise, nurses' awareness of patient participation is important as they play various roles in the entire hemodialysis process [10, 18]. An important factor that can contribute to patient compliance during hemodialysis treatment is the hemodialysis nurse's attitude toward patient participation [19]. Walker et al. [20] evaluated the influence of the dialysis nurse as high as that of the nephrologist in the decision-making process for the patient's choice of renal replacement therapy. In the literature on the role of nephrology specialists in renal replacement therapy, dialysis nurses want to be involved in shared decision-making [21]. However, in South Korea, realistic hospital medical systems and diverse nursing situations exclude nurses from shared medical decision-making, and some nurses also do not know how to participate in shared decision-making [3, 22]. Therefore, it is necessary to identify the factors that affect hemodialysis nurses' awareness of the shared decision-making process for preparing a strategy to improve this decision-making process.

A literature review found that nursing professionalism [14], critical thinking tendency [23], awareness of nursing organizational culture [23], and empathy [5] were reported as the variables correlated with clinical nurses' shared decision-making awareness. Nursing professionalism is a necessary competency in the process of multidisciplinary shared decision-making [14, 24] and provides high-quality nursing through cooperation with various experts in the clinical field, and this allows nurses to perform efficient work [16, 25]. Furthermore, through the establishment of correct professionalism, patients are not excluded from decisions about treatment, but the right to know and autonomy are respected so that they can actively exercise their right to self-determination [10, 26]. Empathy refers to the necessary skills and abilities to understand and alleviate the suffering of others [24]. Empathy has a positive effect on the development of interpersonal relationships, conflict resolution, and facilitated communication [5, 27]. High empathy can provide relief and lower anxiety to patients [5, 28], and can show careful understanding that considers their emotional state and nonverbal expression by identifying their strengths and limitations [29]. It was found that 82% of patients receiving hemodialysis treatment chose empathy as the main factor in forming a therapeutic relationship between patients and nurses [14]. In recent years, the clinical decision-making ability is regarded as an essential competency of nurses in the medical field, and the demand for it is also increasing [13, 30, 31]. Since nurses play the role of experts as patients' protectors, responsibility and decision-making ability are required of them [27]. When faced with various dilemmas related to human dignity and ethical situations on the medical site, nurses make clinical decisions based on moral behavior and critical thinking [32].

**Figure 1:**
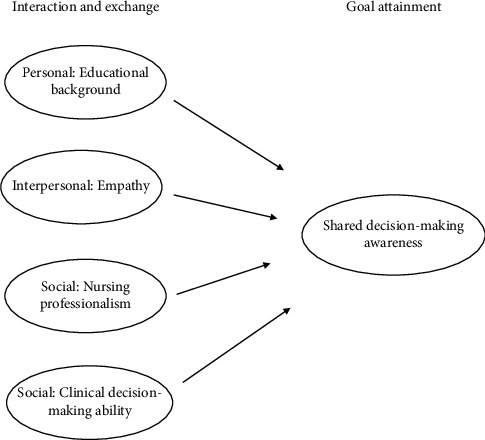
Theoretical framework of this study.

This study uses the King [33] goal attainment theory as its theoretical framework along with a review of previous literature (Figure 1). King's goal attainment theory states that in order to achieve a goal, interaction and exchange of members' perceptions, opinions, and actions are necessary. There are personal, interpersonal, and social levels of interaction and exchange. In order to achieve the goal of shared decision-making awareness, this study reflected the personal level as educational background, the interpersonal level as empathy, and the social level as nursing professional and clinical decision-making ability in interaction and exchange. Therefore, in this study, general characteristics (educational background), empathy, nursing professionalism, and clinical decision-making ability were selected as leading variables that are likely to affect the shared decision-making awareness of hemodialysis nurses (Figure 1). The purpose of this study was to examine the relationships among nursing professionalism, empathy, and clinical decision-making ability and the factors influencing the shared decision-making awareness in hemodialysis nurses.

## 2. Methods

### 2.1. Design, Sample, and Settings

A cross-sectional descriptive design was employed. This study was conducted with nurses working in hemodialysis centers at tertiary general hospitals, general hospitals, and private clinics that operate hemodialysis centers located in Seoul and Gyeonggi-do. The criteria for selecting the study subjects are as follows: (1) hemodialysis nurses with at least one year of work experience and (2) those who agreed to participate in this study. The number of subjects was calculated by using the G^*∗*^power 3.1.9.4 sample number calculation program [34]. Considering the significance level (*α*) = 0.05, power (1-*β*) = 0.80, multiple regression median effect size = 0.15, and 13 independent variables, the minimum number of subjects was 131, and the dropout rate was 10%. A total of 145 subjects responded, and all responses were sufficient, so 145 copies (100%) were finally analyzed.

### 2.2. Instrumentation

Based on a literature review and previous research, a set of general characteristics of the study participants included age, marital status, religion, educational level, total clinical career, hemodialysis career, hospital type, position, and job satisfaction. This consisted of a total of 9 items.

The nursing professionalism scale developed by Yeun et al. [25] was used. This scale consists of 29 questions with 5 subdomains: 9 questions on professional self-concept, 5 on nursing professionalism, 8 on social awareness, 3 on nursing independence, and 4 on nursing practice. Each item is made on a Likert 5-point scale, ranging from 1 point for “strongly disagree” to 5 points for “strongly agree,” and reverse calculations were made for opposite content. A high score means that the nursing professionalism was formed positively, and the score range was 29–145. At the time of development, the Cronbach's *α* of the scale was 0.92, and in the study of Cho [12], the Cronbach's *α* was 0.90, while the Cronbach's *α* value of 0.92 was used in this study.

The empathy scale developed by Lee [35] and modified and supplemented by Lee and Seomoon [36] was used to measure the scale of empathy. It consists of 17 questions with 3 subdomains: 8 questions on communication skills, 5 on sensitivity, and 4 on insights. Each item is made on a Likert 5-point scale, ranging from 1 point for “strongly disagree” to 5 points for “strongly agree,” with higher scores indicating a higher empathy. The score range was 17–85. At the time of scale development, Cronbach's *α*, which refers to reliability, was 0.91, in the study of Seon [37], Cronbach's *α* was 0.89, and in this study, Cronbach's *α* was 0.84.

The clinical decision-making ability scale developed by Jenkins [38] and adapted by Baek [39] was used to measure clinical decision-making ability, and it consists of a total of 40 questions. The scale includes four subdomains: examination of alternatives and options, review of values and goals, examination of information, harmonization of new information, and assessment and reassessment of conclusions. Each subdomain consists of 10 questions. Each item is made on a Likert 5-point scale, ranging from 1 point for “strongly disagree” to 5 points for “strongly agree,” with higher scores indicating a higher clinical decision-making ability. The score range was 40–200. At the time of development proposed by Jenkins [38], Cronbach's *α*, the reliability of this scale, was 0.83, Cronbach's *α* in the study of Baek [39] was 0.77, and Cronbach's *α* in the study of Jang [30] was 0.85. In this study, Cronbach's *α* was 0.72.

The shared decision-making awareness scale developed by Jo [22] was used to measure the level of shared decision-making awareness. It consists of a total of 34 questions with 7 subdomains: 9 questions on information sharing, 7 on establishment of a support system, 5 on duty of explanation, 4 on autonomy, 3 on catching timing, 3 on family participation, and 3 on respect for personality. Each item is made on a Likert 5-point scale, ranging from 1 point for “strongly disagree” to 5 points for “strongly agree,” with higher scores indicating a higher awareness. Score range was 34–170. At the time of tool development, Cronbach's *α*, an indicator of reliability, was 0.80, and in the study of Noh [28], Cronbach's *α* was 0.95. In this study, Cronbach's *α* was 0.94.

### 2.3. Ethical Considerations

This study was conducted after obtaining approval from the Institutional Review Board (IRB no. H-2111-190-1277) of S University Hospital, and the permission from hospital institutes was obtained through the meeting with an explanation. The anonymity and confidentiality of the study were explained to the research subjects, and the study was conducted with the voluntary participation of the study participants who submitted written consent. Researchers explained that the survey will not be used for any purpose other than research and participation can be discontinued at any time during the survey, and even if participation is refused, there will be no disadvantages. Also, the results of the collected questionnaires were managed only by the researcher and were discarded according to the method set by the IRB after the study was completed.

### 2.4. Data Collection

The duration of data collection was from May to July, 2022. Researchers visited and explained the purpose and contents of this study to the tertiary general hospital, hospital, or private clinic where hemodialysis rooms were operated in Seoul and Gyeonggi-do. After sharing the research description and the URL of the Google survey through which one can participate, study participants fully understood the purpose of the study and voluntarily accessed the shared Google survey URL, followed the consent process, and then responded to the survey. The finished survey using a self-reporting questionnaire was collected by online, and they were managed by the authors. The time taken to finish the questionnaires was around 20–25 minutes.

### 2.5. Data Analysis

IBM SPSS version 26.0 (IBM Corp., Armonk, NY, USA) statistical software program analyzed the data from this study. The descriptive statistics using frequency, percentage, mean, and standard deviation analyzed the general characteristics of the study participants and the levels of study variables. The independent *t*-test, ANOVA, and Scheffe post hoc test analyzed the differences in professionalism, empathy, clinical decision-making ability, and shared decision-making awareness according to the general characteristics of the study participants. Pearson's correlation coefficient analyzed the correlations between shared decision-making awareness and related factors. Multiple linear regression statistics analyzed and examined the factors influencing shared decision-making awareness. The statistically significant level of a *p* value was less than 0.05.

## 3. Results

The general characteristics of the study participants are shown in Table 1. As for the age distribution of hemodialysis nurses, 53 (36.6%) were under the age of 35, and 40 (27.6%) were over the age of 45. The average age was 38.89 years old. In terms of marital status, 99 (68.3%) were married; in terms of religion, 83 (57.2%) were nonreligious; and in terms of academic background, 76 people (52.4%) graduated from a four-year university, accounting for the largest proportion. Less than 10 years of clinical experience was most common with 52 (35.9%), and the average clinical experience was 14.36 years. In terms of hemodialysis nurse experience, less than 5 years was the most frequent with 55 (37.9%), and the average experience was 8.21 years. As for the type of hospital where they worked, tertiary general hospital accounted for the highest number with 82 (56%), and 117 (80.7%) were general nurses, and 97 (66.9%) responded that they were satisfied with their current workplace (Table 1).

The levels of shared decision-making awareness, nursing professionalism, empathy, and clinical decision-making ability are presented in Table 2. The mean score for shared decision-making awareness was 147.16, which indicates a high shared decision-making awareness when compared to the median value (102 points) of the score range (34–170). The mean score of nursing professionalism was 102.87, which indicates a slightly low nursing professionalism when compared to the median value (104 points) of the score range (68–140). Their mean score for empathy was 66.68, which indicates a low empathy when compared to the median value (69.5 points) of the score range (54–85). The mean score for clinical decision-making ability was 138.26, which indicates a low clinical decision-making ability when compared to the median value (142.5 points) of the score range (120–165) (Table 2).

Correlations among the study variables are shown in Table 3. Shared decision-making awareness had statistically significant, positive relations with nursing professionalism (*r* = 0.309, *p* < 0.001), empathy (*r* = 0.422, *p* < 0.001), and clinical decision-making ability (*r* = 0.395, *p* < 0.001). The higher the level of nursing professionalism, empathy, and clinical decision-making ability, the higher the shared decision-making awareness (Table 3).

The factors influencing shared decision-making awareness are shown in Table 4. Factors influencing shared decision-making awareness were tested on 145 hemodialysis nurses. Data were collected through questionnaires and shared decision-making awareness among the general characteristics and educational level, which showed a significant difference and were used as predictive variables. Nursing professionalism, empathy, and clinical decision-making ability, which were independent variables that showed statistically significant differences in Pearson's correlation analysis, were also input as predictor variables, and shared decision-making awareness was set as a dependent variable. The collected data were analyzed by using SPSS 26.0. Moreover, there was no outlier larger than the absolute value of 3 when diagnosing cases, so all cases were analyzed with the input method.

First, as a result of testing the regression analysis, all assumptions were satisfied. Durbin–Watson was 1.790, which satisfied Tabachnick [40] criterion of 1.5–2.5. There was no autocorrelation of errors. Furthermore, the correlation coefficients between independent variables were all less than 0.8. As a result of testing multicollinearity by using tolerance limits and VIF values, there was no problem with multicollinearity between variables in which the tolerance limits were less than 0.1 or the VIF values were greater than 10. Then, as a result of analyzing the influence by using Cook's D plot, there was no individual with a value of 1.0 or more among the 145 nurses. In the residual analysis, the linearity and normality of the errors were confirmed with a pictogram, a normal P-P plot of regression standardized residuals, and a normal distribution table. Homoscedasticity was also confirmed as the scatter plot between the standardized residual of the dependent variable and the independent variable did not have a specific distribution, but spread evenly around 0.

After analyzing the regression model, it was found to be significant (*F* = 11.73, *p* < 0.001). The adjusted coefficient of determination (AdjR^2^) was 0.23, showing an explanation power of 23%. The factor that had the greatest influence on the hemodialysis nurse's shared decision-making awareness was empathy (*β* = 0.250, *p*=0.012), followed by clinical decision-making ability (*β* = 0.226, *p*=0.008) and the educational level (*β* = 0.154, *p*=0.046) (Table 4).

## 4. Discussion

The correlation between various study variables and factors influencing shared decision-making awareness was examined. Nursing professionalism had no direct influence on shared decision-making awareness. This is consistent with the results of previous studies [5, 23]. This is thought to be due to the result that the independence area of nursing was the lowest in the subcategories of nursing professionalism. This is because the relationship between doctors and nurses in the medical field is not a horizontal one, but a hierarchical relationship, which limits the ability of nurses to independently make decisions and fulfill their roles [8, 11, 16]. However, when nursing professionalism is established, patients can actively exercise their right to self-determination in the decision-making process during treatment by ensuring the patients' right to know and autonomy in the shared decision-making process [26, 27]. High nursing professionalism raises awareness of ethical decision-making so that nurses can become protectors in the decision-making process of vulnerable subjects, ensuring their participation in the shared decision-making [14, 41]. Through this development, we could confirm that nursing professionalism is a variable correlated with shared decision-making awareness, as in the results of previous studies [5, 12]. As nursing professionalism is constantly developed and can be improved through training, advancement through the development and application of educational programs in clinical practice is required [16, 23]. In clinical settings, when patients are hospitalized in an emergency room and suddenly begin hemodialysis, most patients are not provided with information about the choice of dialysis method and are unable to proceed with shared decision-making [10, 24]. A hemodialysis nurse with a high level of professionalism respects the patient's right to know, provides information that has not been provided, and shows a facilitator who helps patients choose a dialysis method appropriate for their lifestyle through shared decision-making in situations where dialysis must be maintained [12, 14].

The influence of empathy had the greatest effect on shared decision-making awareness. This supports the results of previous studies [5, 28]. Empathy ability was identified as a factor influencing the interpersonal relationship formation of nursing students [42, 43] and facilitated the communication ability of psychiatric nurses [29, 44]. Empathy works as an influencing factor in shared decision-making awareness because this process can form a therapeutic relationship among the patient, family, and healthcare provider, and is performed jointly by forming a positive interpersonal relationship [13, 27]. Moreover, empathy affects communication ability [45], and is a competency required of nurses to understand and care for patients [41, 45], which is consistent with the competency required in the shared decision-making process. The results of previous studies [5, 24] showed that nurses with high education and age showed high empathy, which is partially consistent with the results of this study. Since empathy is a capability that is enhanced by experiential training as well as educational training [44], it is considered that this result emerges because the understanding and experience of the subject could increase as age increases. Therefore, it is necessary to provide practical education and training to improve empathy and enhance shared decision-making awareness. Systemic efforts to reduce the turnover of experienced nurses with high empathy will also be required. Hemodialysis nurses with high empathic ability due to long clinical experience form therapeutic relationships with dialysis patients, enabling patients to make choices with trust in situations of therapeutic choice, and play the role of psychological supporter in the shared decision-making process [13].

Finally, the clinical decision-making ability of hemodialysis nurses was identified as a factor that influences shared decision-making awareness. Applying critical thinking to clinical practice can improve clinical decision-making ability through efficient and prudent responses [31], and make clinical decisions through ethical behavior and critical thinking [32]. Also, critical thinking is an influencing factor that strengthens shared decision-making awareness [23]. Considering these points, clinical decision-making ability could affect the shared decision-making awareness. However, it is difficult to make a direct comparison since there is no previous study on the relationship between these two factors, so further studies will be needed. Among the subdomains of the clinical decision-making ability, evaluation and reevaluation of consequences had the highest score, while the search for alternatives or options had the lowest score, which is consistent with the results of previous studies [31]. Since there are many emergency situations due to hemodynamic instability during hemodialysis, it is difficult to spend time to investigate and select alternatives, such as emergency room nursing, and the patient's condition and outcome are evaluated after dealing with the emergency [28]. Accordingly, the evaluation and reevaluation of consequences scored the highest [32]. The area of search for alternatives or options for various problems is an important part of clinical decision-making, as well as the evaluation of results. Thus, education and training with respect to considering and selecting alternatives for each situation are required. During the process of hemodialysis, many emergency situations occur due to hemodynamic instability, and in such situations, hemodialysis nurses need high clinical decision-making ability [27, 32]. After the emergency situation is resolved, the process and results are evaluated to prepare alternatives to prevent and deal with hemodynamic instability for the patient during the next dialysis.

### 4.1. Study Limitations

This study is limited in the scope of sampling, targeting only hemodialysis nurses working in hemodialysis centers located in Seoul and Gyeonggi-do. Moreover, it is difficult to generalize the results of the study because only hemodialysis nurses who agreed to the survey were included even within the sampling range. Above all, since there is a large difference in the hospital environment where dialysis nurses work, reflection of this is very important, and this may be the limitation of this study.

## 5. Conclusions

In conclusion, nursing professionalism, empathy, and the clinical decision-making ability of hemodialysis nurses were significantly correlated with shared decision-making awareness, and empathy was the most influential factor in shared decision-making awareness as a result of the regression analysis. Awareness was influenced by the clinical decision-making ability and educational level. This study can provide basic data for the development of an intervention program that can improve the shared decision-making awareness among hemodialysis nurses, and can contribute to patient satisfaction and self-care enhancement through active implementation of shared decision-making in the field by improving shared decision-making awareness.

## 6. Implication for Nursing Management

Based on this study, hemodialysis nurses are necessary to improve their empathy and clinical decision-making ability toward hemodialysis patients, whose number is rapidly increasing, while also increasing shared decision-making awareness and promoting patient participation in the process. Ultimately, this will improve the quality of clinical nursing for hemodialysis patients by promoting self-treatment, safe dialysis, and stable management of various chronic diseases, thereby improving their quality of life. Creating an environment to improve shared decision-making awareness of hemodialysis nurses and related training would be essential. Therefore, nursing managers need to pay attention to factors influencing shared decision-making awareness of hemodialysis nurses. Furthermore, it is necessary to develop an intervention program to raise shared decision-making awareness and conduct an experimental study to verify its effectiveness. This study is significant because it could provide basic data to prepare strategies to improve the shared decision-making awareness of hemodialysis nurses.

## Figures and Tables

**Table 1:** General characteristics of the study participants (*n* = 145).

Variables	Categories	*N*	%	Mean (SD)
Age (years)	<35	53	36.6	38.89 (8.56)
	35∼44	52	35.9	
	45≤	40	27.6	

Marital status	Unmarried	46	31.7	
	Married	99	68.3	

Religion	Not have	83	57.2	
	Have	62	42.8	

Educational level	Associate	35	24.1	
	Bachelor	76	52.4	
	Above master	34	23.4	

Total clinical career (years)	<10.0	52	35.9	14.36 (8.4)
	10.0∼19.9	48	33.1	
	20.0≤	45	31.0	

Hemodialysis career (years)	<5.0	55	37.9	8.21 (6.35)
	5.0∼9.9	40	27.6	
	10.0≤	50	34.5	

Hospital type	Tertiary general hospital	82	56.6	
	General hospital	25	17.2	
	Private clinic	38	26.2	

Position	General nurse	117	80.7	
	Charge nurse	28	19.3	

Job satisfaction	Satisfaction	97	66.9	
	Dissatisfaction	48	33.1	

**Table 2:** Levels of shared decision-making awareness, nursing professionalism, empathy, and clinical decision-making ability (*n* = 145).

Variables	Mean (SD)	Min	Max	Mean (SD)	Range
Shared decision-making awareness	147.16 (11.74)	114	170	4.33 (0.35)	34∼170

Sharing information		3.33	5.00	4.46 (0.40)	1∼5
Constructing system		3.00	5.00	4.19 (0.41)	
Explanation duty		3.20	5.00	4.54 (0.40)	
Autonomy		2.25	5.00	4.16 (0.51)	
Capturing time		3.00	5.00	4.38 (0.40)	
Participation of family		2.67	5.00	4.27 (0.50)	
Human respect		2.00	5.00	4.14 (0.58)	

Nursing professionalism	102.87 (13.01)	68	140	3.55 (0.45)	68∼140

Self-concept of the profession		2.00	5.00	3.58 (0.53)	1∼5
Social recognition		1.75	4.75	3.30 (0.58)	
Professionalism of nursing		2.40	5.00	3.88 (0.48)	
Role of nursing service		1.75	5.00	3.77 (0.51)	
Originality of nursing		1.33	5.00	3.26 (0.67)	

Empathy	66.68 (5.67)	54	85	3.92 (0.33)	54∼85

Communication		2.63	5.00	3.89 (0.38)	1∼5
Sensitivity		3.00	5.00	4.07 (0.42)	
Insight		2.75	5.00	3.81 (0.43)	

Clinical decision-making ability	138.26 (8.67)	120	165	3.46 (0.22)	120∼165

Evaluation and reevaluation of consequences		2.56	4.67	3.64 (0.40)	1∼5
Canvassing of objectives and values		2.90	4.60	3.59 (0.29)	
Search for information and unbiased assimilation of new information		3.00	4.40	3.56 (0.31)	
Search for alternatives or options		2.40	3.70	3.02 (0.23)	

**Table 3:** Correlations among study variables (*n* = 145).

Variables	Shared decision-making awareness *r* (*p*)	Nursing professionalism r (*p*)	Empathy *r* (*p*)	Clinical decision-making ability *r* (*p*)
Shared decision-making awareness	1			
Nursing professionalism	0.309 (<0.001^*∗*^)	1		
Empathy	0.422 (<0.001^*∗*^)	0.607 (<0.001^*∗*^)	1	
Clinical decision-making ability	0.395 (<0.001^*∗*^)	0.355 (<0.010^*∗*^)	0.473 (<0.010^*∗*^)	1

^*∗*^ *p* < 0.05.

**Table 4:** Factors influencing shared decision-making awareness (*n* = 145).

Variables	B	SE	*β*	*t*	*p*	Tolerance	VIF
Constant	61.15	14.41		4.24	<0.001		
Educational level	2.61	1.30	0.154	2.01	0.046^*∗*^	0.91	1.10
Nursing professionalism	0.04	0.08	0.042	0.45	0.651	0.62	1.61
Empathy	0.52	0.20	0.250	2.54	0.012^*∗*^	0.55	1.82
Clinical decision-making ability	0.31	0.11	0.226	2.69	0.008^*∗*^	0.76	1.32

Durbin–Watson's *d* = 1.790 (1.679 ≤ *d* ≤ 1.788), Adj*R*^2^ = 0.23, *F* = 11.73, *p* < 0.001^*∗*^							

VIF = variance inflation factor; ^*∗*^*p* < 0.05

## Data Availability

The data that support the findings of this study are available from the corresponding author upon reasonable request.
